# Pioneering the Integration of Artificial Intelligence in Medical Oral Board Examinations

**DOI:** 10.7759/cureus.52318

**Published:** 2024-01-15

**Authors:** Satoshi Hanada, Yuri Hayashi, Sudhakar Subramani, Kokila Thenuwara

**Affiliations:** 1 Anesthesia, University of Iowa Hospitals and Clinics, Iowa City, USA; 2 Department of Anesthesiology and Intensive Care Medicine, Osaka University Graduate School of Medicine, Suita, JPN

**Keywords:** anesthesiology, medical education technology, chatgpt-4, oral board examination, artificial intelligence (ai)

## Abstract

We evaluated the use of ChatGPT-4, an advanced artificial intelligence (AI) language model, in medical oral examinations, specifically in anesthesiology. Initially proven adept in written examinations, ChatGPT-4's performance was tested against oral board sample sessions of the American Board of Anesthesiology. Modifications were made to ensure responses were concise and conversationally natural, simulating real patient consultations or oral examinations. The results demonstrate ChatGPT-4's impressive adaptability and potential in oral board examinations as a training and assessment tool in medical education, indicating new avenues for AI application in this field.

## Editorial

Artificial intelligence (AI) has recently made remarkable strides in medicine [1-3], leaving indelible footprints, particularly with the emergence of ChatGPT [4,5]. The advanced language model, ChatGPT-4, has demonstrated a striking aptitude for emulating human conversation since its launch in early 2023. This AI's prowess is notable in written examinations [6-8], such as the U.S. Medical Licensing Examination [9] and specialty board certification written examinations, including the Royal College of Anaesthetists written examination [10].

**Figure 1:**
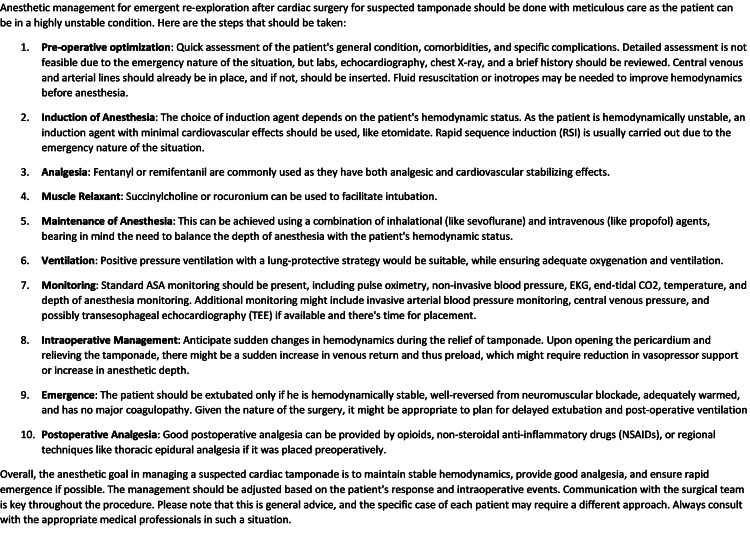
Response from ChatGPT-4 to the following selected question from the ABA oral board sample sessions: A 65-year-old man underwent an uncomplicated CABG 16 hours earlier and was extubated four hours ago. In the past hour, his BP fell from 110/70 to 70/50, and the CVP rose from 8 to 22 mmHg. If tamponade is suspected and mediastinal exploration is required, how would you provide anesthesia?

Nevertheless, a hitherto unexplored territory is the potential of ChatGPT in oral examinations. We delved into the performance of ChatGPT-4 in oral board examinations, which evaluate an examinee’s clinical judgment, adaptability to unanticipated clinical changes, and proficiency in organizing and presenting information. Remarkably, when published American Board of Anesthesiology (ABA) sample sessions [11] were presented to ChatGPT-4, it generated answers that met, or even exceeded, the passing criteria for all questions, as judged by two ABA board-certified anesthesiologists (SH and KT). The initial responses were thorough and comprehensive; however, they were lengthy and lacked a natural conversational tone (Figure 1).

**Figure 2:**
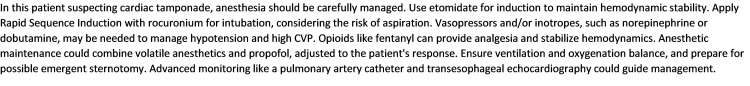
The subsequent response from ChatGPT-4 to the following instruction: Answer within 100 words.

Thus, we implemented constraints, instructing AI to limit responses to a word count, as if a person had paused to think and respond in the allotted time (Figure 2).

**Figure 3:**
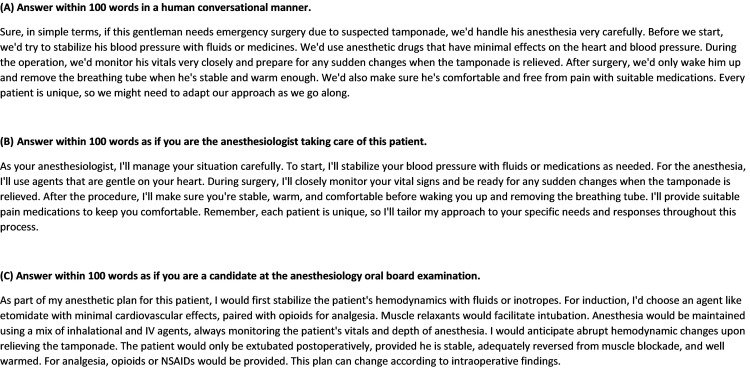
The subsequent responses from ChatGPT-4 to the following instruction: (A) Answer within 100 words in a human conversational manner. (B) Answer within 100 words as if you are the anesthesiologist taking care of this patient. (C) Answer within 100 words as if you were a candidate for the anesthesiology oral board examination.

We then added instructions to emulate a conversational tone and to simulate an anesthesiologist's consultation with a patient or a board examination scenario. This resulted in concise, information-dense answers that adhere closely to human conversation patterns, such as in a real oral examination (Figures 3A-3C).

AI has access to a wealth of information; still, it requires clear instructions to provide the expected response. Our expertise lies in anesthesiology; thus, we chose an anesthesia example. However, this model could be applied to diverse medical specialties. The responses provided by AI are truly impressive, complete with clinical decision-making and the reasoning behind it. This technology could serve as a tool to test both the validity and reliability of question design and also to assist candidates in preparing for oral board examinations. Moreover, the adaptability of ChatGPT-4 suggests its potential role in teaching and preparing medical professionals for real-world clinical scenarios, thereby enhancing their communication and decision-making skills. These facets are new frontiers in the application of AI in medical education and examination, paving the way for further advancements in this rapidly evolving field.
